# Late onset hemopericardium with cardiac tamponade from minor blunt chest trauma – a case report

**DOI:** 10.1002/ccr3.216

**Published:** 2015-02-02

**Authors:** Petronilla N Tabansi, Barbara E Otaigbe

**Affiliations:** Department of Pediatrics and Child Health, University of Port Harcourt/University of Port Harcourt Teaching HospitalPort Harcourt, Rivers State, Nigeria

**Keywords:** Cardiac tamponade, late onset, minor chest trauma

## Abstract

Hemopericardium with cardiac tamponade following minor blunt trauma is a rare, life-threatening condition in children. Without high index of suspicion, diagnosis and intervention may be delayed as the link between the trauma and illness may be missed. We present a 12-year-old female in Nigeria, and highlight challenges in diagnosis.

## Introduction

Pericardial effusion in children can occur from a variety of causes, more commonly infectious and inflammatory conditions affecting the pericardium; and in some cases from penetrating chest trauma 1. Pericardial effusion from blunt chest trauma is rare especially when such trauma is minor, such that patients may not report or volunteer the information without much probing by the doctor 2,3. The delay in the pediatric population evokes an extensive list of differential diagnoses. With any added history of trauma, the effusion should be considered secondary to hemorrhage 4. Cardiac tamponade is a life-threatening condition and without intervention, affected patients go into cardiogenic shock and death 1. The primary abnormality is a rapid or slow compression of all the cardiac chambers as a result of increasing intrapericardial pressure due to the accumulating effusion which in time exceed the stretch limit of the pericardium 5. Consequently, myocardial diastolic compliance becomes reduced limiting cardiac inflow. Critical tamponade is a form of cardiogenic shock. Tachypnea and dyspnea on exertion that progresses to air hunger at rest are key symptoms. Affected patients also present with tachycardia, distant heart sounds, raised jugular venous pressure (JVP), and hypotension. A key diagnostic finding is pulsus paradoxus – conventionally defined as an inspiratory systolic fall in arterial pressure of 10 mmHg or more during normal breathing – and may often be palpable in muscular arteries 5–7. Without intervention, there is progression to cardiogenic shock and death.

## Case

C.N. is a 12-year-old female secondary school student who presented to the Children Emergency Ward of the University of Port Harcourt Teaching Hospital with a 2-week history of progressive weakness, generalized body swelling which started at the feet but later involved the abdomen, and 1-week history of progressive difficulty in breathing, even at rest. Parents say that she had been in a boarding school at the time of onset, and in relative good health prior to this episode of illness. There was no history of fever, headache, rash, or other constitutional symptoms. Past medical history revealed no previous hospital admissions or serious illness. Examination findings showed that she was in severe respiratory distress, severely pale with bilateral pitting pedal edema, and mildly dehydrated. Respiratory system examination revealed tachypnea (40 cpm), stony dull percussion notes in the middle, and lower lung zones bilaterally. She also had deceased vocal and tactile fremitus in these areas. Breath sound was vesicular with reduced intensity. There were few basal crepitations bilaterally. Cardiovascular system (CVS) examination showed a low pulse volume, pulsus paradoxus, raised jugular venous pressure (JVP) of 11 cm, H_2_O, hypotension (BP – 60/30 mmHg), and tachycardia of 120 bpm. The apex beat could not be palpated and the first and second heart sounds were very distant. Abdominal examination showed a tender hepatomegaly of 7 cm below the right costal margin, with mild ascites demonstrable by shifting dullness. Other systemic examinations were normal.

**Figure 1:**
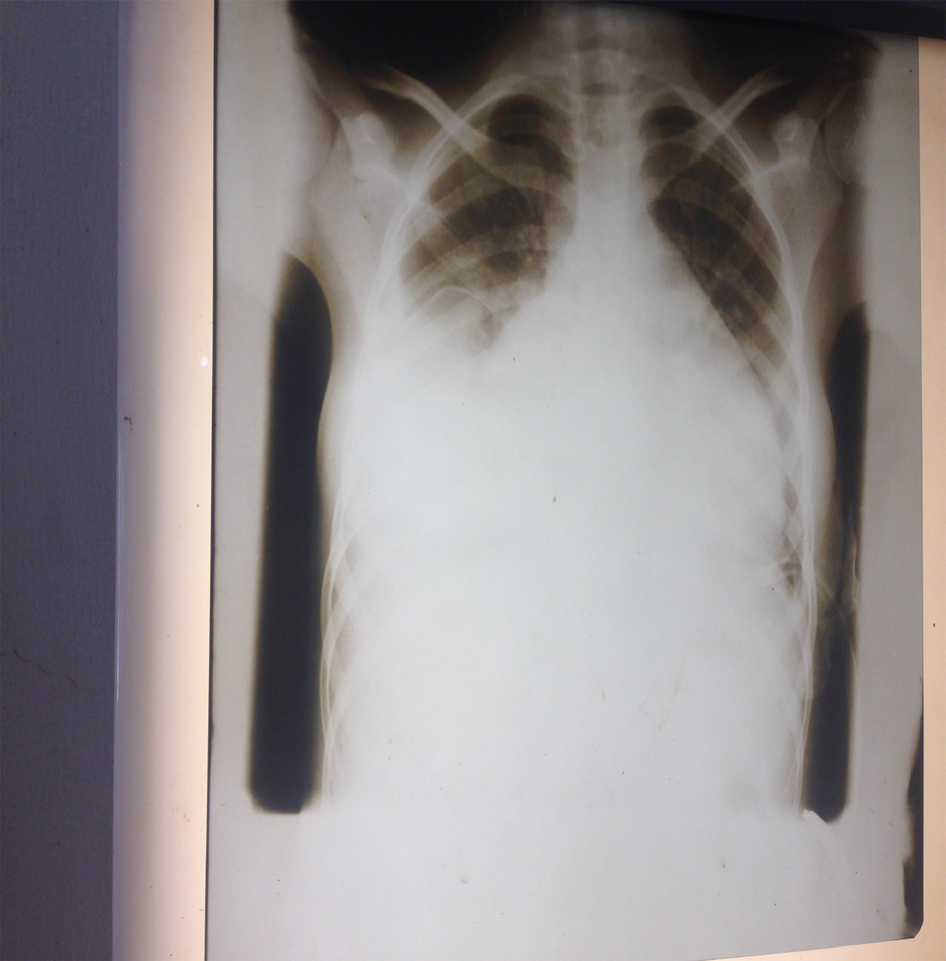
Enlarged cardiac silhouette due to pericardial effusion.

**Figure 2:**
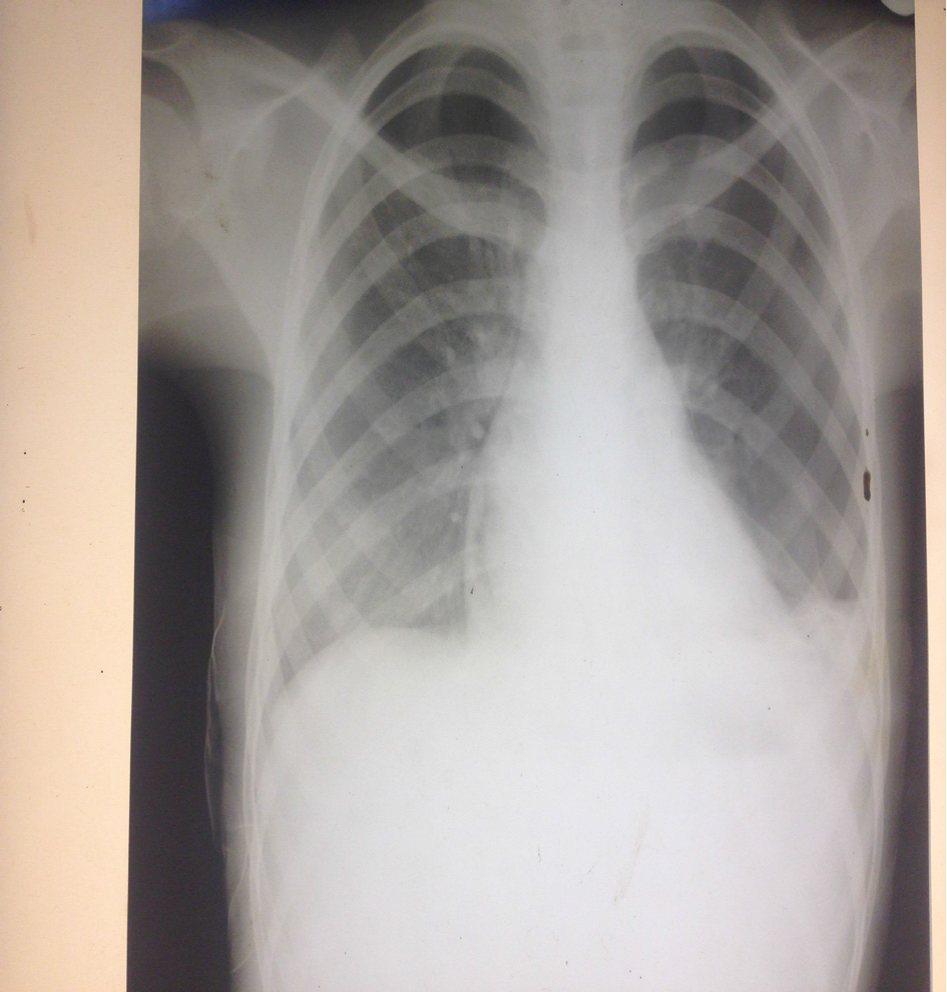
Normal cardiac silhouette after pericardiocentesis.

An initially diagnosis of congestive cardiac failure with bilateral pleural effusion and large pericardial effusion, probably due to viral myocarditis and pericarditis to rule out tuberculosis was made. She was then commenced on antiheart failure drug therapy and bed rest in cardiac position. A chest radiograph showed evidence of bilateral pleural effusion and a massive cardiac silhouette (Figure 1 & 2). However, a pleural fluid aspiration yielded clear fluid on both sides with chemical analysis suggestive of a transudate. A Mantoux text was also negative and full blood count (FBC) and blood film were normal except for low packed cell volume (PCV) of 23% (hemoglobin of 7.6 g/dL).

A pericardial aspiration was then performed which yielded frank blood which was nonclotting. The sample was sent for extensive analysis including full blood count, blood film for cytology, blood microscopy, culture and sensitivity, and microscopy and culture for acid fast bacilli, which all yielded negative results.

Further questioning revealed that she had had a minor chest trauma (she hit her sternum on the edge of her bed rail) while scampering from her dormitory mistress, about 3 weeks prior to the onset of symptoms. At the time, she had a mild chest pain which relieved spontaneously, and thought nothing of it thereafter. She also had no prior bleeding tendencies, no history suggestive of an autoimmune disorder or exostosis. There was also negative family history of bleeding diathesis.

**Figure 3:**
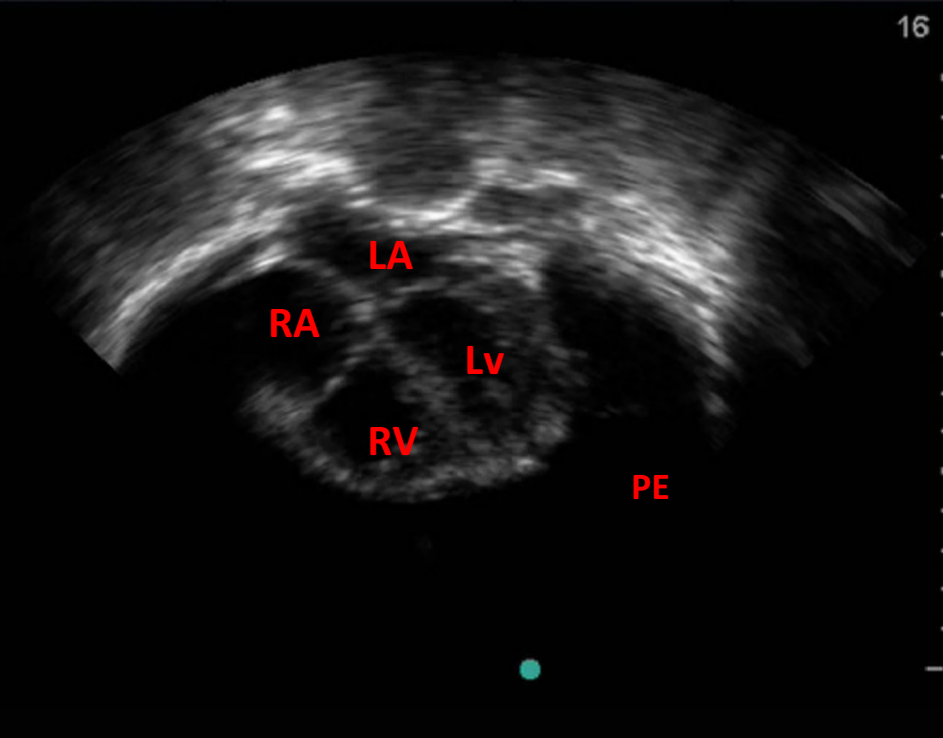
Apical Four Chamber view showing large pericardial effusion PE, RA, Right atrium; RV, Right ventricle; LA, Left Atrium; LV, Left ventricle.

An echocardiography showed massive pericardial effusion with shrunken atria and ventricles and poor diastolic function (Figure 3). There was also swinging motion of the heart and compression of the ventricular cavities. A definitive diagnosis of massive hemorrhagic pericardial effusion from minor blunt chest trauma, with cardiac tamponade, congestive cardiac failure and impending cardiogenic shock was made.

She immediately had pericardiocentesis using a pericardial catheter inserted via the substernal approach. A total of 1.5 L of nonclotting blood was drained. Patient had immediate relief of respiratory distress, and the pulse volume improved, with increase in blood pressure to 100/60 mmHg, even before the procedure was over. She was subsequently transfused with one unit of whole blood and had rapid recovery, and was discharged after few days of observation in good health. She has been on follow-up at the Pediatric Cardiology clinic for over 3 years now and is well.

## Discussion

Hemopericardium with cardiac tamponade after a blunt chest wall trauma in children is a very rare but life-threatening condition 8–10. Various case reports have linked minor blunt chest trauma with delayed hemopericardium as seen in the index patient 6–11. Because the causes of pericardial disease and thus cardiac tamponade are diverse, careful and thorough clinical history and examination are necessary to clinch the diagnosis 11. The cardiac damages and/or their complications are often missed because they may develop several weeks following the injury;^8^ and as such, the causal relation with the traumatic event is less evident 9. This is what occurred in this case as the history of trauma was not volunteered until specifically asked for, because the patient did not link it to her illness since it occurred weeks earlier. This led to a delay in diagnosis and institution of appropriate treatment before overt signs of cardiac tamponade became evident, as has been reported in other cases 10,11.

Tachycardia and jugular venous distention are key signs of cardiac tamponade 5–7, and our patient had both a tachycardia of 120 bpm and raised JVP of 11 cmH_2_O. The raised JVP is due to compression of the right atrium causing back pressure to the jugular veins 5–7. Other key signs which were also evident in the patient include tachypnea and dyspnea on exertion that progressed to dyspnea even at rest. She also had demonstrable pulsus paradoxus as there was a fall in her inspiratory systolic blood pressure of 15 mmHg, which was also palpable in the brachial artery as a comparatively feeble pulse during inspiration. Pulsus paradoxus has been reported to be a key diagnostic finding in cardiac tamponade 5,12. The patient also had very distant heart sounds on auscultation due to the insulating effects of the pericardial fluid, and to reduced cardiac function. This has also been reported elsewhere in literature 13.

The patient could be said to have been in evolving cardiogenic shock as her blood pressure was quite low at 60/30 mmHg, with small volume pulse, although her extremities were not yet cold and clammy nor was there peripheral cyanosis. Patients with cardiac tamponade are often reported to be in cardiogenic shock 14, due to the cardiac compression compromising cardiac filling and stroke volume. It is possible that any further delay in her diagnosis and intervention would have resulted in a fatal outcome.

**Figure 4:**
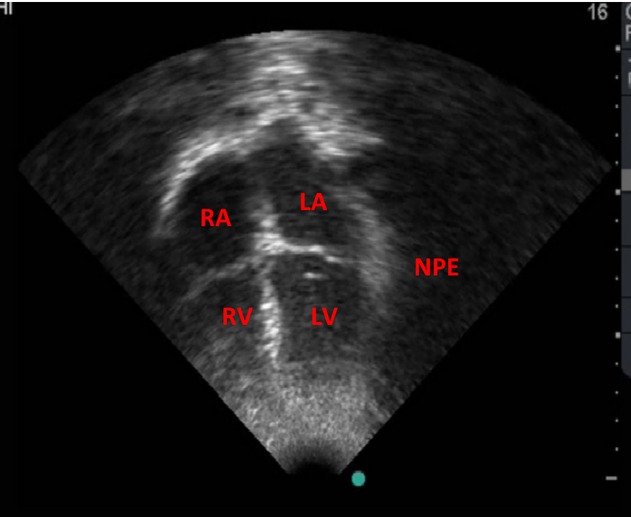
Apical Four Chamber view after pericardiocentesis showing expansion of the Chambers and absence of the pericardial effusion. (NPE, No pericadial effusion).

Relief of the tamponade via pericardiocentesis resulted in a rapid decompression, and expansion of her cardiac chamber which was observed during echocardiography (Figure 4); and resulted in marked clinical improvement. She had relief of her dyspnea, blood pressure increased toward normal and her pulse became full volume. This intervention is life saving and is due to the improvement in the diastolic compliance such that the diastolic filling and hence stroke volume and cardiac output returned to normal. She has since remained in good health and participates actively in physical activities.

## Conclusion

The report concludes that late onset hemopericardium with cardiac tamponade following a minor blunt chest wall trauma can occur in children, and is difficult to recognize before overt signs of cardiac tamponade has developed. Also, a causal relationship between the minor trauma and hemopericardium may be missed (due to the time lapse) if the doctor does not probe further. Thus, a high index of suspicion, thorough history and prompt intervention is necessary to avert mortality from consequent cardiogenic shock.

## Conflict of Interest

None declared.
